# Sepsis-associated sarcopenia: Emerging insights into mechanisms and therapeutic approaches

**DOI:** 10.1016/j.jointm.2026.03.005

**Published:** 2026-05-18

**Authors:** Xiujun Chang, Fan Zeng, Jun Wang, Zhaoxuan Guo, Rongan Liu, Yu Lei, Jiajia Li, Fuxun Yang, Xiaoxiu Luo, Chun Pan, Xiaobo Huang, Yunping Lan

**Affiliations:** 1 Department of Intensive Care Unit, Sichuan Academy of Medical Sciences and Sichuan Provincial People’s Hospital, Chengdu, Sichuan, China; 2 College of Medicine and Life Sciences of Chengdu University of Traditional Chinese Medicine, Chengdu, Sichuan, China; 3 School of Medicine, University of Electronic Science and Technology of China, Chengdu, Sichuan, China

**Keywords:** Sarcopenia, Sepsis, Mitochondrial, Nutritional support, Rehabilitation

## Abstract

As medical treatment advances, a growing proportion of sepsis patients survive from their initial critical illness. However, excessive treatment and diminished quality of life after discharge raise substantial concerns. Sarcopenia affects 40%–70% of sepsis survivors, manifesting as flaccid weakness in the extremities and diaphragm. This condition typically resolves within weeks or months, but in some cases, recovery does not occur and may last for 2 years after intensive care unit discharge. Consequently, sepsis patients with sarcopenia face an unfavorable long-term influence, and they also consume considerable medical resources. The mechanisms and treatments of sepsis-associated sarcopenia (SASP) are increasingly recognized as research priorities. This review provides a comprehensive analysis of molecular mechanisms, measuring parameters, and therapeutic approaches for SASP.

## Introduction

According to the latest European Working Group on Sarcopenia in Older People (EWGSOP2) guidelines, the diagnostic criteria for sarcopenia are: the presence of low muscle strength suggests possible sarcopenia; if accompanied by low muscle quantity or quality, a diagnosis can be confirmed; if further accompanied by low physical performance, it is considered severe sarcopenia.^[^^1^^]^ Based on categories, sarcopenia can be classified into primary and secondary types. Primary sarcopenia is a kind of muscular atrophy linked to aging. It is characterized by gradual degeneration resulting in the loss of skeletal muscle mass, quality, strength, and usefulness.^[^^2^^]^ It is estimated that the prevalence of sarcopenia ranges from 5% to 13% in people who are 60–70 years old, and it rises to 11% to 50% in those who are over 80.^[^^3^^,^^4^^]^ Secondary sarcopenia may be classified into physiological variables and pathological conditions. Physiological sarcopenia largely results from insufficient protein consumption, reduced physical exercise, and extended immobilization. Pathological factors involve various systemic diseases, including neurodegenerative syndromes such as Alzheimer’s disease, cancer-induced cachexia, inflammatory disorders, including sepsis, and the beginning of sarcopenia is associated with chronic conditions such as diabetes and chronic obstructive pulmonary disease (COPD).^[^^5^^]^

Intensive care unit-acquired weakness (ICU-AW) is fundamentally characterized by sarcopenia,^[^^6^^]^ and sarcopenia is pathologically characterized by a reduction of fibers, reduced cross-sectional area and muscle mass, diminished or absent muscle strength, and impaired coordination of muscle contraction and relaxation, ultimately leading to muscular dysfunction.^[^^7^^,^^8^^]^ Sepsis, prevalent among critically ill patients, has been shown to cause both rapid muscle deterioration and enduring damage to muscle tissue.^[^[9], [10], [11], [12]^]^ Sarcopenia poses two significant risks in people with sepsis. Initially, sarcopenia is significantly linked to extended ventilator use,^[^^13^^]^ which extends the duration of hospitalization. If not adequately managed, it can lead to severe clinical outcomes, including high mortality rates.^[^^14^^]^ Second, the prognosis for sepsis survivors after discharge is unfavorable,^[^^15^^,^^16^^]^ characterized by substantial reductions in exercise capacity, muscular strength, and a diminished overall quality of life.^[^^17^^,^^18^^]^ These findings contributed to elevated readmission rates^[^^14^^]^ and increased 1-year^[^^19^^]^ and long-term mortality following discharge.^[^^20^^]^ Currently, research into the molecular mechanisms and therapeutic approaches for sepsis-associated sarcopenia (SASP) remains largely confined to the preclinical stage, with a conspicuous scarcity of high-quality clinical evidence. This fundamental research-clinical gap is consequently compounded by the absence of reliable diagnostic tools and effective therapeutic alternatives in clinical practice. This review will critically synthesize current knowledge on SASP pathogenesis, measuring parameters and interventions, and conclude with a critical appraisal of research limitations and future directions to provide insightful perspectives.

## Methods and Search Strategy

A systematic search strategy was employed to comprehensively collect relevant literature. A search strategy was constructed using the MeSH thesaurus in the PubMed database. Sepsis/septic shock was used as a subject heading, with “Sepsis” and “Shock, septic” as MeSH terms, and their free-text terms included “Bloodstream infection” and “Septic shock.” Sarcopenia and muscle atrophy were used as subject headings, with “Sarcopenia” and “Muscle Atrophy” as MeSH terms. Free-text terms for “Sarcopenia” included: “Muscle weakness” and “Muscle loss.” Free-text terms for “Muscle atrophy” included: “Muscular atrophy,” “Muscle wasting,” and “Muscle damage.” Searches were conducted in the PubMed, Embase, and Web of Science databases, with the search period spanning from January 1, 2015, to October 30, 2025. Inclusion criteria were: (1) Study subjects were clinical patients or animal models diagnosed with sepsis/septic shock; (2) Study content was related to sarcopenia or muscle atrophy; (3) Literature published in English. Exclusion criteria were: (1) Clinical trial registrations, conference abstracts, or letters to the editor; (2) Incomplete experimental data; (3) Ex vivo or cell-level studies; (4) Duplicate results. The methodological quality of the included studies was independently assessed by two reviewers using specific design tools, including the Cochrane Risk of Bias Assessment Tool (RoB-2.0) for randomized controlled trials (RCT),^[^^21^^]^ the Newcastle-Ottawa scale (NOS) for clinical cohort studies (CS),^[^^22^^]^ and the SYRCLE’s risk of bias tool for preclinical animal studies.^[^^23^^]^ Any disagreements during the quality assessment process are resolved through discussion or arbitration by a third adjudicator. The results from all included studies were synthesized.

## Mechanisms

The normal function of skeletal muscle does not exist in isolation. Skeletal muscle is not only a faithful executor of central commands but also a powerful signal transmitter. It engages in continuous, dynamic dialogue with the endocrine system and metabolic organs such as the gut to jointly maintain the body’s metabolic homeostasis and exercise capacity. Dysfunction at any level can lead to skeletal muscle abnormalities. SASP results from multi-level dysregulations—from organelles to distant organs—interacting to form a self-sustaining vicious cycle (Figure 1). Sepsis acts as the “trigger,” simultaneously igniting destructive processes at multiple levels. Mitochondria, the “powerhouse” of the cell, initiate an intracellular bioenergetic crisis, while endoplasmic reticulum stress (ERS) triggers apoptotic signaling. At the cell level, protein degradation pathways are systematically activated, including the ubiquitin–proteasome system (UPS), the autophagic-lysosomal pathway (ALP), calpains, myostatin (MSTN), and programmed cell death; while anabolic and repair pathways are suppressed, manifested by the shutdown of insulin-like growth factor-1 (IGF-1)-mediated protein synthesis and the dysfunction of muscle satellite cells (MuSCs). Within the tissue microenvironment, inflammatory cytokines mount a sustained chemical assault, and simultaneously, innervation is disrupted, leaving the muscle “isolated and helpless.” Ultimately, the systemic endocrine system (appetite, thyroid hormone, and glucocorticoid) and inter-organ communication (the gut–muscle axis) are profoundly disrupted. This locks the people with sepsis into a global metabolic state of “high catabolism and low anabolism,” leading to the continuous depletion of muscle tissue and the development of difficult-to-reverse sarcopenia.

**Figure 1:**
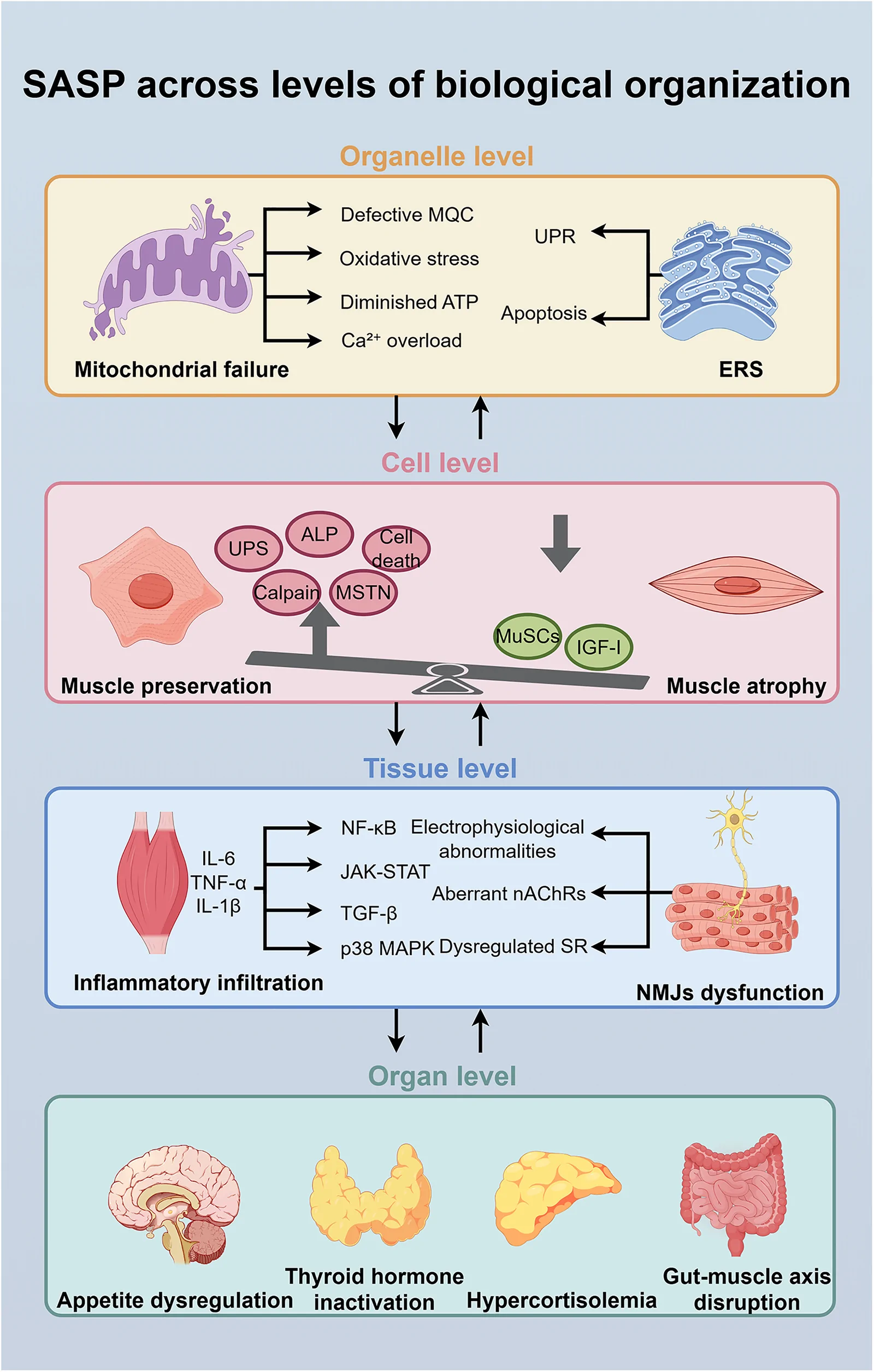
SASP across levels of biological organization. The pathogenesis of SASP manifests as a multi-level, multi-dimensional, and progressively amplifying cascade process, encompassing interactions across subcellular organelles, cells, tissues, and organs. At the organelle level, it primarily involves mitochondrial failure (including defective MQC, activation of oxidative stress, diminished ATP synthesis, and mitochondrial Ca^2+^ overload) and ERS (activating the UPR and inducing myofiber apoptosis). At the cell level, protein degradation (involving activation of UPS, ALP, calpain, and cell death pathways) surpasses protein synthesis (activation of MSTN, inhibition of MuSCs, and IGF-1 pathways), leading to atrophy of preservative muscle. At the tissue level, characteristics include skeletal muscle inflammatory infiltration (IL-6, TNF-α, and IL-1β activating NF-κB, JAK-STAT, TGF-β, and p38 MAPK signaling pathways, promoting skeletal muscle atrophy) and NMJs dysfunction (electrophysiological abnormalities, aberrant expression of nAChRs, and dysregulated SR). At the organ-interaction level, mechanisms involve dysregulation of hypothalamic appetite control, inactivation of thyroid hormone, hypercortisolemia, and disruption of the muscle–gut axis. ALP, Autophagy-lysosome pathway; ATP, Adenosine triphosphate; Ca^2+^, Calcium; ERS, Endoplasmic reticulum stress; IGF-I, Insulin-like growth factor I; IL, Interleukin; JAK-STAT, Janus kinase-signal transducer and activator of transcription; MQC, Mitochondrial quality control; MSTN, Myostatin; MuSCs, Muscle satellite cells; nAChRs, Nicotinic acetylcholine receptors; NF-κB, Nuclear factor kappa-B; NMJs, Neuromuscular junctions; P38 MAPK, p38 mitogen-activated protein kinase; SASP, Sepsis-associated sarcopenia; SR, Sarcoplasmic reticulum; TGF, Transforming growth factor; UPR, Unfolded protein response; UPS, Ubiquitin–proteasome system.

However, current mechanistic research faces important limitations and evidence gaps. Due to the challenges of obtaining muscle biopsies from septic patients, only a few human studies have reliably confirmed three core pathological changes: UPS activation,^[^^24^^]^ inflammatory cell infiltration,^[^^25^^]^ and ERS.^[^^26^^]^ Most proposed mechanisms derive from rodent models and in vitro studies, with a scarcity of high-quality human data. SASP represents a rapid pathological cascade initiated by organelle-level metabolic failure, leading to systemic muscle degradation within a short timeframe and progressing to an irreversible state through multi-organ crosstalk. The therapeutic window is narrow and reversal is challenging. Current understanding of human pathophysiology still relies heavily on animal models, and a substantial gap remains in developing precise and effective clinical interventions. This evidence disparity constitutes a key obstacle that must be overcome in future translational research.

### Organelle dysfunction

At the organelle level, SASP does not result from an inadequate energy supply alone.^[^^27^^]^ Recent studies have increasingly highlighted the pathological accumulation of dysfunctional mitochondria.^[^^28^^]^ These organelles undergo both quantitative and qualitative changes: morphologically abnormal mitochondria accumulate owing to defective mitochondrial quality control (MQC), while functionally, they demonstrate heightened oxidative stress, diminished adenosine triphosphate (ATP) synthesis, and calcium (Ca²⁺) overload (Figure 2). Together, these alterations severely impair bioenergetic output and reduce muscle strength. In parallel, persistent ERS and the activated unfolded protein response (UPR) further aggravate the atrophy of muscle fibers.

**Figure 2:**
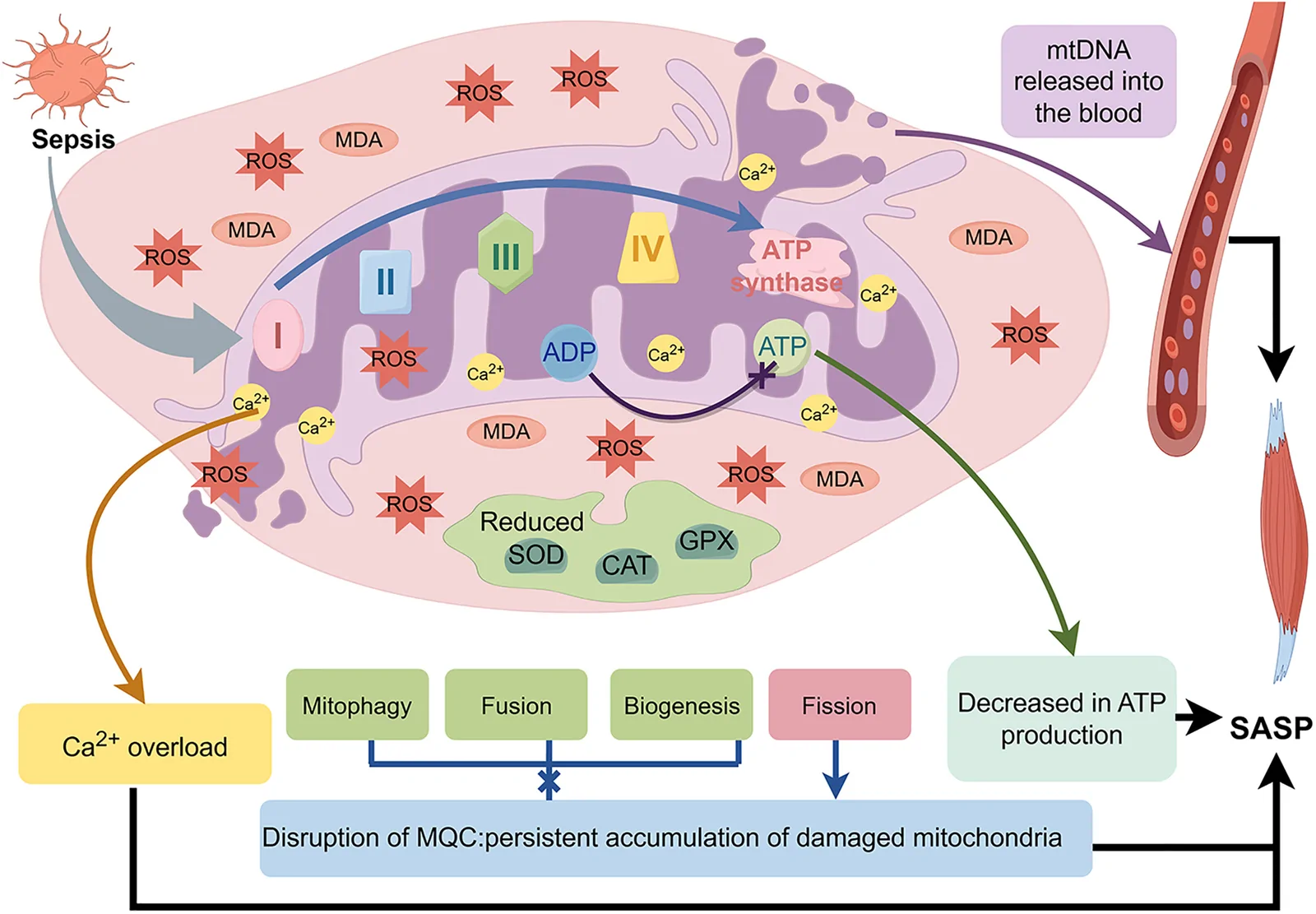
Mitochondrial failure: disrupted quality control and bioenergetic crisis. Under septic conditions, skeletal muscle mitochondria exhibit significant abnormalities in quantity, quality, and function. The regulation of MQC is disrupted, as mitophagy, fusion, and biogenesis are suppressed while fission is excessively activated, resulting in the persistent accumulation of damaged mitochondria. Functionally, these mitochondria demonstrate markedly enhanced oxidative stress—characterized by elevated ROS and MDA alongside reduced antioxidants like SOD, CAT, and GPX—as well as decreased ATP production and Ca^2+^ overload. Furthermore, damaged mitochondria released mtDNA into the blood, exacerbating systemic inflammatory responses. Together, these alterations delineate a core pathological process of disrupted MQC and bioenergetic crisis. ADP, Adenosine diphosphate; ATP, Adenosine triphosphate; Ca^2+^, Calcium; CAT, Catalase; GPX, Glutathione peroxidase; MDA, Malondialdehyde; MQC, Mitochondrial quality control; mtDNA, Mitochondrial DNA; ROS, Reactive oxygen species; SASP, Sepsis-associated sarcopenia; SOD, Superoxide dismutase.

#### Mitochondrial failure: disrupted quality control and bioenergetic crisis

Sepsis animal models exhibit substantial alterations in both the quantity and structural integrity of skeletal muscle mitochondria. The proportion of healthy mitochondria decreases by more than 35%,^[^^29^^]^ with limb muscles experiencing a more pronounced reduction.^[^^30^^]^ Dysfunctional mitochondria progressively accumulate over time. By day 14, sepsis survivors retain only 7.5% normal mitochondria in muscle fibers, a level markedly lower than the 16.8% observed during the acute phase on day 4.^[^^31^^]^ Ultrastructural analyses reveal widespread morphological abnormalities, including mitochondrial swelling,^[^^32^^]^ disrupted cristae,^[^^33^^]^ “onion-like” whorled cristae formations,^[^^34^^,^^35^^]^ membrane rupture, matrix vacuolization,^[^^36^^]^ and overall expansion.^[^^37^^]^ The presence of relatively intact mitochondrial membranes in some regions suggests a localized reparative capacity. Under physiological conditions, MQC maintains functional integrity through biogenesis, fusion-fission dynamics, and autophagic clearance. In sepsis, this system becomes globally impaired: peroxisome proliferator-activated receptor-gamma coactivator 1-α (PGC-1α) suppression disrupts biogenesis^[^^27^^,^^38^^,^^39^^]^ reduced fusion,^[^^29^^]^ and enhanced fission fragments the mitochondrial network^[^^30^^,^^33^^]^ and impaired mitophagy permits the persistent accumulation of damaged organelles.^[^^36^^,^^40^^,^^41^^]^ MQC failure not only compromises cellular ATP production and contractile function but also exacerbates systemic inflammation via mitochondrial DNA (mtDNA) release into the blood, which activates innate immune pathways.^[^^34^^]^

Oxidative stress plays a central role in sepsis-induced mitochondrial dysfunction in skeletal muscle, characterized by excessive reactive oxygen species (ROS) generation and compromised antioxidant capacity.^[^^38^^,^^42^^]^ Key antioxidant enzymes—superoxide dismutase (SOD),^[^^43^^,^^44^^]^ catalase (CAT), and glutathione peroxidase (GPX)—show markedly reduced activity, with particular downregulation of the mitochondrial isoforms SOD2^[^^39^^]^ and GPX4.^[^^45^^]^ Concurrently, the sirtuin 1/nuclear factor erythroid 2-related factor 2/heme oxygenase-1 (Sirt-1/Nrf-2/HO-1) signaling axis,^[^^46^^,^^47^^]^ a master regulator of antioxidant responses, is significantly suppressed, leading to accumulation of the lipid peroxidation marker malondialdehyde (MDA).^[^^43^^,^^48^^,^^49^^]^ Notably, oxidative stress responses display muscle-type heterogeneity; limb muscles such as the soleus and extensor digitorum longus exhibit greater susceptibility than the diaphragm.^[^^50^^]^ Although diaphragmatic SOD2 expression can decline by up to 70% with concurrent MDA elevation,^[^^29^^]^ its intrinsically stronger antioxidant baseline may confer partial protection. While ROS could theoretically promote proteolysis through oxidation of proteolytic pathway components, direct evidence establishing ROS as an upstream trigger of muscle protein breakdown in sepsis remains limited.

ATP synthesis relies critically on mitochondrial functional integrity. During sepsis, impaired mitochondrial membrane potential,^[^^51^^]^ reduced electron transport chain (ETC) complex I–IV activities,^[^^27^^,^^31^^,^^33^^]^ and increased proton leak collectively^[^^52^^]^ diminish ATP output.^[^^37^^,^^53^^,^^54^^]^ This bioenergetic deficit directly contributes to muscle weakness and impaired mobility. Mitochondrial injury appears persistent, even during recovery, when muscle mass normalizes, ultrastructural defects—including disrupted cristae, vacuolization, and persistently low complex I activity—often remain,^[^^35^^]^ potentially underlying long-term weakness in sepsis survivors.

Dysregulated mitochondrial Ca^2+^ handling represents another key pathological mechanism. Downregulated mitochondrial Ca^2+^ uptake 1 (MICU1) expression coupled with upregulated mitochondrial Ca^2+^ uniporter (MCU) expression induces pathological Ca^2+^ overload even under resting conditions, further compromising skeletal muscle mitochondrial function.^[^^55^^]^

Although mitochondrial dysfunction is recognized as a cornerstone of SASP, the field faces important translational challenges. Current mechanistic evidence derives almost exclusively from animal studies, requiring rigorous validation in human sepsis. More fundamentally, whether mitochondrial dysfunction initiates net proteolysis or merely reflects atrophy sequelae remains unresolved due to insufficient direct evidence. Future studies should prioritize human tissue validation, establish definitive causal links from structural defects to functional decline, and develop mitochondria-targeted therapies to bridge the translational gap.

#### ERS: UPR and proteostasis disruption

ERS and its associated UPR are critically involved in the pathogenesis of SASP.^[^^56^^]^ Animal studies show that sepsis markedly upregulates ERS marker expression in skeletal muscle, with these dynamic changes temporally aligning with the expression of various atrophy-related genes, suggesting a close link between ERS and muscle wasting.^[^^57^^]^ Genetic deletion of C/EBP homologous protein (CHOP), a key pro-apoptotic effector in ERS signaling, not only mitigates UPS-mediated protein degradation but also lowers the phosphorylation levels of signal transducer and activator of transcription 3 (STAT3) and mothers against decapentaplegic homolog 3 (Smad3), thereby partially reversing muscle atrophy.^[^^58^^]^ Ultrastructural analyses further reveal pronounced endoplasmic reticulum dilation accompanied by unfolded protein accumulation and a sharp rise in ERS markers during the acute phase, such as at 48 h, whereas these alterations largely resolve by the recovery phase, for example, by day 7.^[^^59^^]^ These findings suggest that a moderate UPR aids proteostatic recovery, whereas persistent or excessive ERS promotes net protein loss and programmed cell death in myofibers via CHOP-dependent apoptosis and signaling pathways, including STAT3 and Smad3. However, this field faces significant limitations, as only a single clinical study has thus far confirmed ERS marker upregulation in the rectus abdominis muscle of septic patients,^[^^26^^]^ while the remaining mechanistic evidence comes entirely from preclinical models, creating a substantial gap in human tissue validation. Consequently, the precise function of ERS/UPR in human sepsis-induced sarcopenia requires further clarification, and future research should prioritize clinical studies to address this translational deficit.

### Collapse of myofibrillar proteostasis

At the cell level, proteostasis rapidly collapses, accompanied by a broad activation of protein degradation pathways such as the UPS, the ALP, calpain activation, and multiple programmed cell death pathways.^[^^60^^]^ Concurrently, protein synthesis is suppressed through elevated MSTN levels, exhaustion of MuSCs, impaired myoblast differentiation, and blocking protein synthesis-related pathways, ultimately leading to irreversible net protein loss and a failure of muscle regeneration (Figure 3).

**Figure 3:**
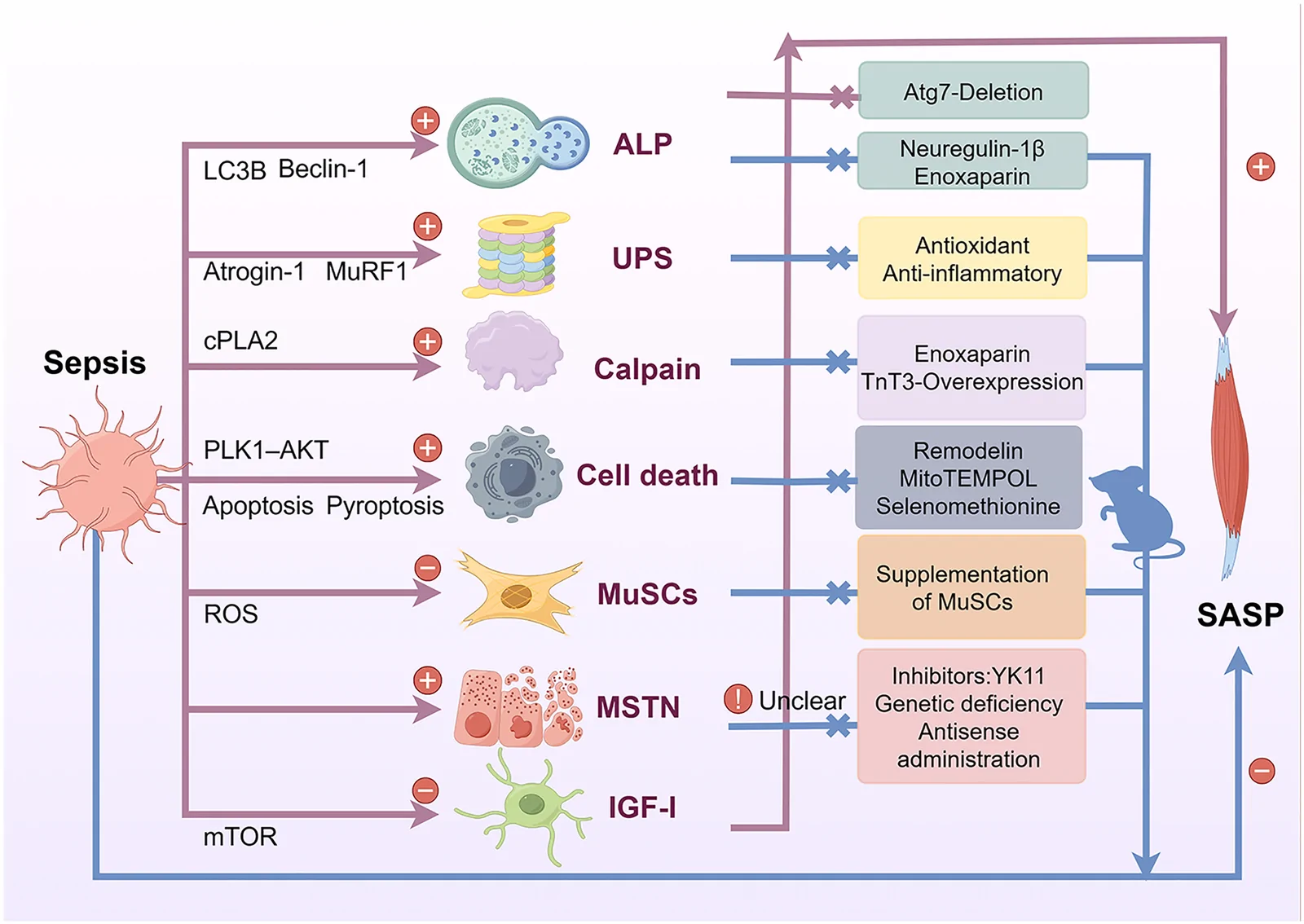
Collapse of myofibrillar proteostasis. Skeletal muscle fibers are primarily composed of proteins. Under septic conditions, multiple signaling pathways act synergistically to induce net protein degradation in muscle fibers, including ALP activation via LC3B and Beclin-1, a process whose complex regulatory role is highlighted by the inhibition observed with Atg7 deletion. This degradation is further driven by UPS activation via Atrogin-1 and MuRF1, calpain activity enhancement through cPLA2, and apoptosis induction via the PLK1-AKT signaling pathway and other myofiber death pathways such as pyroptosis. Concurrent activation of MSTN, increased ROS release suppressing MuSCs function, and mTOR pathway-mediated inhibition of IGF-1 also contribute. Collectively, these mechanisms lead to the collapse of myofibrillar proteostasis. Reflecting these pathological mechanisms, basic research has shown that SASP can be ameliorated by reversing the corresponding pathways: neuregulin-1β and enoxaparin improve SASP by inhibiting the ALP, while antioxidant and anti-inflammatory strategies do so by inhibiting the UPS. Enoxaparin and TnT3 overexpression improve SASP by inhibiting calpain, and remodelin and MitoTEMPOL improve SASP by inhibiting apoptosis, whereas selenomethionine improves SASP by inhibiting pyroptosis. Supplementation of MuSCs also alleviates SASP. However, findings regarding MSTN inhibition remain inconsistent, as YK11 and antisense MSTN administration ameliorate SASP, but genetic deficiency of MSTN does not confer protection. These results reveal the complex regulatory network underlying the collapse of myofibrillar proteostasis and its potential therapeutic targets. ALP, Autophagy-lysosome pathway; Atg7, Autophagy-related protein 7; Atrogin-1, Muscular atrophy F-box; cPLA2, Calcium-dependent phospholipase A2; IGF-I, Insulin-like growth factor I; LC3B, Microtubule-associated protein 1A/1B-light chain 3; MitoTEMPOL, Inhibitors of apoptosis; MSTN, Myostatin; mTOR, Mammalian target of rapamycin; MuRF1, Muscle RING finger protein 1; MuSCs, Muscle satellite cells; PLK1-AKT, Polo-like kinase 1-protein kinase B; ROS, Reactive oxygen species; SASP, Sepsis-associated sarcopenia; TnT3, Troponin T3;UPS, Ubiquitin–proteasome system; YK11, MSTN inhibitors.

#### Hypercatabolism: systemic activation of protein degradation pathways

Scientific consensus maintains that the UPS has long been regarded as the principal pathway for intracellular protein degradation and remains the most thoroughly characterized mechanism in the development of SASP.^[^^61^^]^ Its activation has been directly confirmed in rectus abdominis muscle biopsies from septic patients.^[^^24^^]^ The system’s degradation efficiency is chiefly regulated by ubiquitin-protein ligases (E3), with the muscle-specific E3 ligases muscular atrophy F-box (Atrogin-1) and muscle RING finger protein 1 (MuRF1) acting as rate-limiting enzymes. Increased expression of these ligases is widely regarded as a key molecular marker of elevated proteolysis in skeletal muscle.^[^^43^^,^[62], [63], [64], [65], [66]^]^ Recent investigations have increasingly focused on the upstream regulatory network controlling UPS activity. Several compounds with antioxidant properties, such as edaravone^[^^46^^]^ and anisodamine,^[^^47^^]^ or anti-inflammatory properties, such as melatonin^[^^67^^]^ and astragaloside IV,^[^^68^^]^ have demonstrated potential in preclinical studies to alleviate SASP by suppressing UPS activity. These observations imply that targeting upstream signaling to reduce proteasomal activity could provide new interventional approaches for limiting sepsis-associated muscle proteolysis. However, this area of research faces significant constraints; current evidence originates almost entirely from animal studies and cellular models, while the target specificity and therapeutic effectiveness of these interventions remain unvalidated in human subjects. Consequently, the efficacy, safety, and clinical translational potential of such strategies must be further assessed through rigorously designed clinical trials.

Under physiological conditions, autophagy sustains cellular homeostasis by eliminating damaged organelles and misfolded proteins, and it facilitates cellular adaptation to nutrient stress by generating energy and substrates via catabolism. In sepsis, however, skeletal muscle experiences severe autophagic dysregulation. Selective autophagic clearance becomes impaired, resulting in considerable accumulation of dysfunctional mitochondria and protein aggregates that directly disrupt myofiber integrity and contractile function.^[^^41^^]^ Muscle-specific deletion of autophagy-related protein 7 (Atg7) exacerbates metabolic dysfunction and increases mortality in septic mice,^[^^69^^]^ while Parkin overexpression improves mitochondrial quality and attenuates SASP.^[^^32^^]^ Conversely, although organelle-specific autophagy is deficient, overall autophagic flux in muscle fibers can be excessively activated,^[^^70^^,^^71^^]^ as reflected by elevated microtubule-associated protein 1A/1B-light chain 3 (LC3B)^[^^72^^]^ and Beclin-1 levels^[^^73^^]^ alongside reduced sequestosome-1 (p62), ultimately provoking excessive protein breakdown and muscle atrophy.^[^^74^^]^ Preclinical studies indicate that pharmacological agents such as neuregulin-1β^[^^75^^]^ and enoxaparin^[^^73^^]^ may alleviate sepsis-associated muscle wasting by counteracting hyperactive autophagy. Substantial controversy persists concerning the temporal pattern and dual functions of autophagy in sepsis-induced muscle atrophy—specifically, when its activity is protective as opposed to detrimental, and whether therapies should aim to stimulate or suppress it. Moreover, all existing mechanistic insights derive from preclinical models, with a conspicuous absence of direct validation in human tissues or clinical trials.

Disruption of calcium homeostasis represents a core pathophysiological event that activates calpain during severe sepsis. As widely expressed cysteine proteases in skeletal muscle, calpains directly promote muscle atrophy by mediating apoptotic signaling pathways and by specifically cleaving contractile-associated proteins. In the septic environment, sustained calpain activation disrupts sarcomeric structural integrity, leading to the disassembly and release of key myofilament components such as actin and myosin.^[^^62^^,^^71^^,^^76^^,^^73^^,^^77^^]^ Studies further indicate that calcium-dependent phospholipase A2 (cPLA2), acting as an upstream regulator of calpains, can induce diaphragmatic dysfunction through calpain-dependent mechanisms.^[^^78^^]^ In experimental sepsis models, administration of calpain inhibitors such as enoxaparin^[^^73^^]^ or overexpression of troponin T3 (TnT3)^[^^62^^]^ has been shown to effectively attenuate muscle atrophy and significantly reduce skeletal muscle protein degradation rates, highlighting the therapeutic potential of targeting this pathway. However, current understanding of calpain’s role in SASP is almost exclusively derived from animal studies, lacking in vivo dynamic validation in human muscle tissues. Although preclinical studies demonstrate the protective effects of calpain inhibitors, their target specificity, optimal dosing timing, and safety profiles in septic patients require systematic evaluation. Furthermore, the crosstalk mechanisms between calpains and other proteolytic pathways, including the UPS and ALP, remain incompletely elucidated, presenting significant challenges for developing precise intervention strategies.

In SASP, programmed cell death and proteolytic systems constitute a complex regulatory network. Beyond the classical UPS and ALP, multiple regulated cell death modalities—including apoptosis, pyroptosis,^[^^63^^]^ ferroptosis, and cuproptosis—contribute to its pathogenesis.^[^^79^^]^ Apoptosis remains the most extensively characterized pathway.^[^^43^^,^^80^^,^^81^^]^ Sepsis directly triggers myofiber apoptosis and impairs regeneration by activating the polo-like kinase 1–protein kinase B (PLK1–AKT) signaling axis.^[^^82^^]^ Corresponding intervention studies demonstrate that inhibitors such as remodelin^[^^48^^]^ and MitoTEMPOL^[^^77^^]^ effectively suppress apoptosis, while selenomethionine^[^^64^^]^ mitigates muscle atrophy by inhibiting pyroptosis. By contrast, the functions of the more recently identified ferroptosis and cuproptosis pathways in SASP remain largely unexplored. Although theoretical frameworks propose that ferroptosis^[^^83^^]^ operates via lipid peroxidation and cuproptosis^[^^84^^]^ through copper-dependent mechanisms, direct experimental evidence from sepsis models is still scarce. Critical features—including activation thresholds, specific molecular markers, and their relative pathological contributions—have yet to be defined. Overall, programmed cell death forms a multilayered but incompletely resolved regulatory network in SASP. Current research reveals a pronounced mechanistic imbalance: whereas the apoptotic pathway has been relatively well-elucidated, evidence for emerging mechanisms such as ferroptosis and cuproptosis relies heavily on bioinformatic predictions or in vitro observations, lacking robust in vivo validation and clinical correlation. This substantial knowledge gap significantly constrains a comprehensive understanding of SASP pathology and the development of targeted therapeutic strategies.

#### Anabolic suppression: failure of repair and regeneration

MuSCs are the principal agents of skeletal muscle regeneration, migrating, proliferating, and terminally differentiating to repair or replace injured myofibers. In the pathological context of sepsis, however, the myogenic differentiation of MuSCs is profoundly disrupted, characterized by a marked suppression of the key transcription factors.^[^^85^^,^^86^^]^ Concurrent disruption of intracellular metabolic homeostasis reduces oxidative phosphorylation efficiency, induces mitochondrial dysfunction,^[^^87^^,^^88^^]^ and promotes excessive reactive ROS accumulation.^[^^86^^]^ These changes collectively diminish the regenerative capacity of MuSCs, thereby accelerating the progression of skeletal muscle atrophy in SASP. While preclinical studies indicate that exogenous MuSCs supplementation can partially restore muscle contractile function by modulating inflammatory cytokines, restoring mitochondrial function, and improving metabolic activity,^[^^89^^]^ all current evidence originates from animal models. The survival rate and myogenic differentiation efficiency of MuSCs within the human septic microenvironment remain uncharacterized, and the safety and efficacy of such therapeutic strategies have not been systematically evaluated in clinical settings. These critical knowledge gaps substantially impede the translation of MuSCs-targeted regenerative therapies from basic research to clinical application.

MSTN, a negative regulatory factor expressed specifically in skeletal muscle, strongly inhibits myocyte proliferation, differentiation, and protein synthesis. Current research shows that the MSTN signaling pathway becomes abnormally activated in animal models of sepsis,^[^^63^^,^^90^^,^^91^^]^ and interventions targeting this pathway—including pharmacological inhibitors such as YK11,^[^^92^^]^ MSTN antisense administration,^[^^91^^]^ and genetic knockout^[^^93^^]^ have been reported to partially mitigate the pathological progression of SASP. Nevertheless, this area of research is characterized by notable contradictions and limitations. For instance, findings from Morel et al.^[^^53^^]^ indicate that MSTN knockout in septic animal models failed to produce the expected anti-atrophy effects a result that conflicts with outcomes from other studies. These discrepancies underscore substantial gaps in understanding the role of MSTN in SASP, which may be affected by variables such as the choice of animal model, intervention timing, and muscle-type specificity. Moreover, all current evidence originates from preclinical studies, leaving the efficacy and safety of MSTN-targeted strategies in human septic patients unverified and thereby impeding their translation into clinical therapy.

The IGF-I signaling pathway is a central regulator of protein synthesis in skeletal muscle. During sepsis, the body develops marked resistance to anabolic signals, characterized by impaired mammalian target of rapamycin (mTOR) signaling ^[^^58^^,^^62^^,^^66^^,^^94^^,^^95^^]^ and a profound suppression of IGF-I biological activity.^[^^72^^,^^96^^,^^97^^]^ Previous research indicates that pharmacologically increasing local IGF-I bioavailability in muscle could partially attenuate the progression of SASP without substantially disrupting systemic metabolic homeostasis.^[^^98^^]^ Nevertheless, this area of investigation faces significant constraints: studies examining the IGF-I pathway’s role and therapeutic targeting in SASP have been limited in recent years, and the available evidence relies entirely on preclinical animal models, lacking validation in human septic patients. Furthermore, the systemic inflammatory environment in sepsis may persistently disrupt downstream IGF-I signaling through multiple mechanisms, making strategies focused exclusively on elevating ligand levels inadequate for effectively reversing impaired protein synthesis.

### Structural and functional disintegration of skeletal muscle

At the tissue level, skeletal muscle undergoes acute inflammatory infiltration and structural disruption. Cytokines amplify catabolic signaling through pathways such as nuclear factor kappa-B (NF-κB), janus kinase (JAK)-STAT, transforming growth factor-β (TGF-β), and p38 mitogen-activated protein kinase (P38 MAPK), accelerating protein breakdown (Figure 4). Furthermore, dysfunction of nicotinic acetylcholine receptors (nAChRs) in the endplate membrane, combined with dysregulated Ca^2+^ handling by the sarcoplasmic reticulum (SR), leads to excitation-contraction uncoupling (Figure 5). This impairment directly contributes to the clinically observed muscle incoordination.

**Figure 4:**
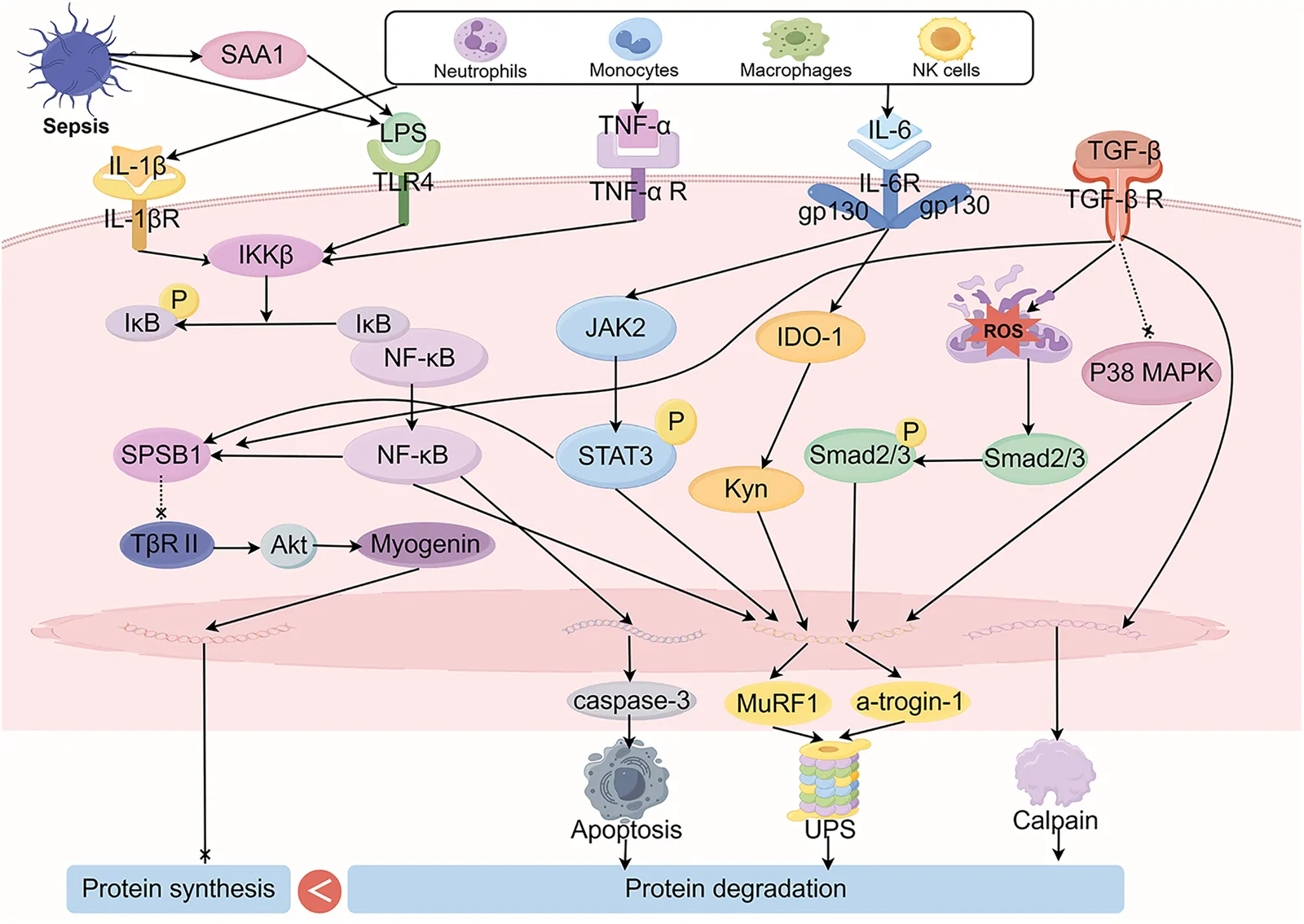
Inflammatory infiltration and amplified catabolic signaling. Sepsis activates innate immune cells (neutrophils, monocytes, macrophages, and NK cells), which release cytokines including IL-1β, TNF-α and IL-6. LPS and these cytokines bind to their cognate receptors on muscle fibers, activating multiple signaling pathways that synergistically drive myofibrillar protein degradation and suppress protein synthesis. The NF-κB pathway, primarily activated by IL-1β, TNF-α, and LPS, promotes downstream myofiber apoptosis via caspase-3 and activates the UPS. IL-6 predominantly activates the JAK-STAT pathway, which also enhances UPS activity. TGF-β stimulates ROS release and promotes UPS via Smad2/3, while also directly activating calpain. Furthermore, IL-6 activates UPS through the IDO-1-Kyn pathway. Together, these pathways collectively accelerate skeletal muscle proteins degradation. In the early phase of sepsis, however, TGF-β suppresses myofiber UPS by inhibiting the p38 MAPK pathway, thereby partially attenuating myofibrillar protein degradation. Concurrently, the JAK-STAT, NF-κB, and TGF-β pathways inhibit the TβRII-Akt-myogenin axis via SPSB1, reducing skeletal muscle protein synthesis. The synergistic action of these inflammatory pathways shifts myofibers toward net protein degradation. Akt, Inhibiting protein kinase B; ALP, Autophagy-lysosome pathway; Atrogin-1, Muscular atrophy F-box; gp130, Glycoprotein 130; IDO-1, Indoleamine 2,3-dioxygenase 1; IKKβ, Inhibitor of kappa B kinase; IL-1β, Interleukin-1β; IL-1β R, Interleukin-1β receptor; IL-6, Interleukin-6; IL-6 R, Interleukin-6 receptor; IκB, Inhibitor of nuclear factor kappa-B; JAK2, Janus kinase 2; Kyn, Kynurenine; LC3B, Microtubule-associated protein 1A/1B-light chain 3; LPS, Lipopolysaccharide; MuRF1, Muscle RING finger protein 1; NF-Κb, Nuclear factor kappa-B; NK, Natural killer; P38 MAPK, p38 mitogen-activated protein kinase; ROS, Reactive oxygen species; SAA1, Acute-phase protein serum amyloid A1; Smad2/3, *Drosophila* mothers against decapentaplegic homolog 2/3; SPSB1, SOCS box protein 1; STAT3, Signal transducer and activator of transcription 3; TGF-β, Transforming growth factor-β; TGF-β R, Transforming growth factor-β receptor; TLR4, Toll-like receptor 4; TNF-α, Tumor necrosis factor-α; TNF-α R, Tumor necrosis factor-α receptor; TβRII, Transforming growth factor-β receptor II; UPS, Ubiquitin–proteasome system.

**Figure 5:**
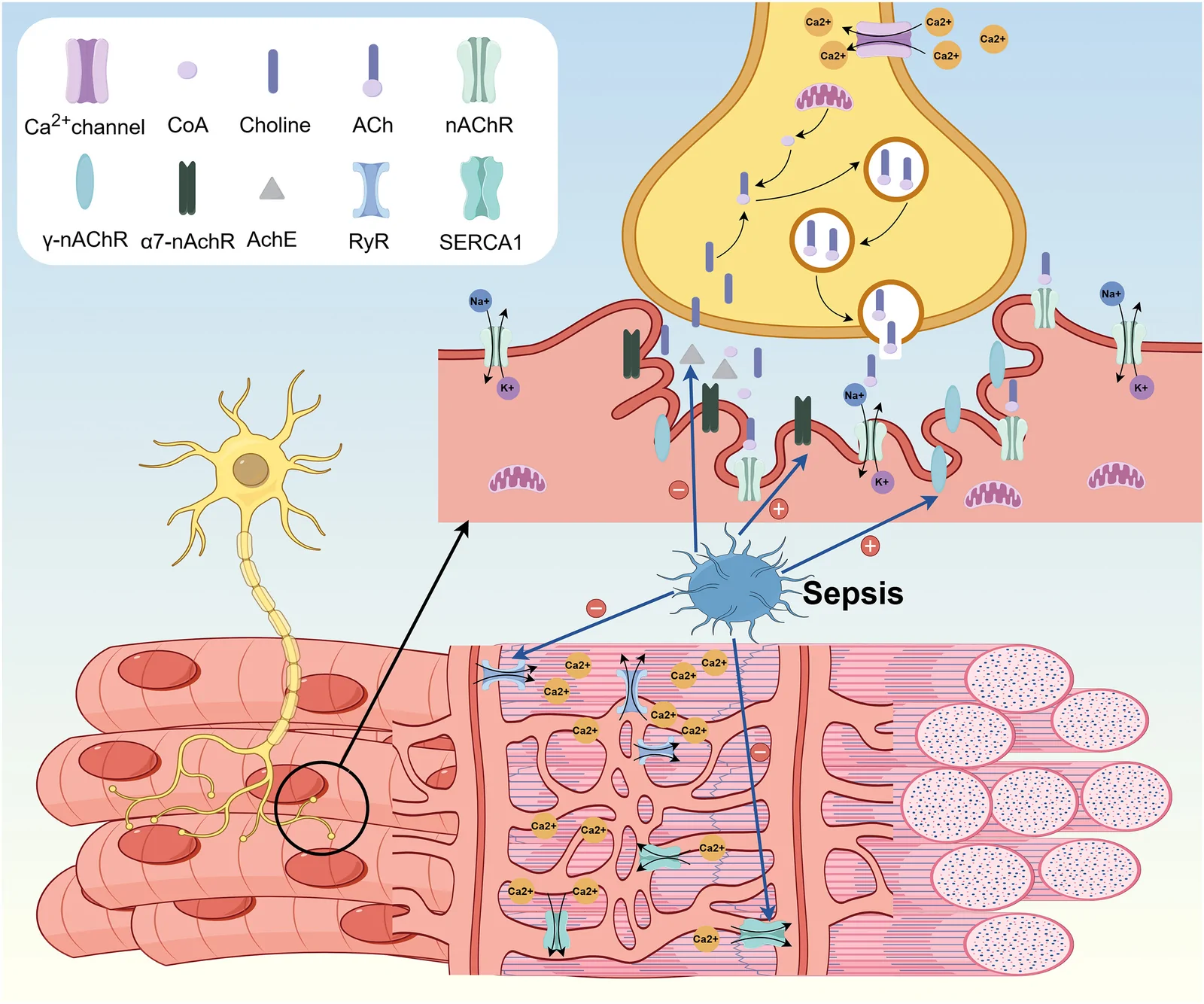
NMJ dysfunction and excitation–contraction uncoupling. Sepsis induces NMJ dysfunction through two primary mechanisms. First, it reduces AChE activity at the postsynaptic membrane while upregulating the expression of aberrant γ-nAChR and α7-nAChR. Second, sepsis suppresses the expression of RyR and SERCA1 in the SR, which disrupts Ca^2+^ release and reuptake and leads to abnormal skeletal muscle contraction. Together, these mechanisms underscore the key pathological roles of NMJ dysfunction and excitation–contraction uncoupling in SASP. ACh, Acetylcholine; AChE, Acetylcholinesterase; Ca^2+^, Calcium; CoA, Coenzyme-A; nAChRs, Nicotinic acetylcholine receptors; NMJ, Neuromuscular junction; RyR, Ryanodine receptor; SASP, Sepsis-associated sarcopenia; SERCA1, Sarco/endoplasmic reticulum Ca^2+^ adenosine triphosphatase 1; α7-nAChRs, α7-type nicotinic acetylcholine receptors; γ-nAChRs, fetal-type nicotinic acetylcholine receptors.

#### Inflammatory infiltration and amplified catabolic signaling

Sepsis patients often endure prolonged disease courses marked by persistent systemic inflammatory responses throughout treatment and progression. This reaction arises from the interplay between invasive pathogens and the host innate immune system, including natural killer (NK) cells, neutrophils,^[^^99^^]^ and macrophages,^[^^100^^]^ which substantially elevates circulating pro-inflammatory cytokines such as interleukin-6 (IL-6), tumor necrosis factor-α (TNF-α), and interleukin-1β (IL-1β).^[^^41^^,^[65], [66], [67], [68]^,^^72^^,^^42^^]^ These mediators disseminate to multiple tissues and organs, including skeletal muscle,^[^^41^^,^^30^^]^ the hypothalamus,^[^^101^^]^ and the gut, where they exacerbate protein catabolism via highly expressed Toll-like receptors (TLRs) on muscle cells.^[^^25^^,^^102^^,^^103^^]^ Clinical studies confirm that septic patients exhibit sustained serum IL-6 elevation^[^^24^^]^ and significant upregulation of TLR2 and TLR4 in skeletal muscle.^[^^25^^]^ Beyond activating the JAK-STAT signaling pathway,^[^^104^^]^ IL-6 promotes SASP progression by inducing the indoleamine 2,3-dioxygenase 1 (IDO-1)-kynurenine (Kyn) pathway.^[^^24^^]^ Consequently, targeting IL-6 has emerged as a key therapeutic strategy to alleviate muscle atrophy.^[^[105], [106], [107]^]^ Animal studies show that IL-6 blockade reduces mitochondrial ROS production, thereby effectively delaying SASP progression.^[^^38^^]^ However, cytokines do not act uniformly as detrimental factors; interleukin-10 (IL-10)^[^^35^^]^ displays protective effects and is elevated in sepsis survival models, underscoring the complexity of inflammatory regulation. Current research in this area remains limited. Most mechanistic evidence derives from animal studies, and the efficacy and safety of IL-6 inhibition in humans await validation through clinical trials. During sepsis, key signaling pathways, including NF-κB, JAK–STAT, TGF-β, and P38 MAPK, undergo persistent activation, collectively driving chronic inflammation in skeletal muscle. These pathways act synergistically to disrupt proteostasis in muscle fibers, shifting the balance toward sustained protein breakdown over synthesis and ultimately leading to progressive muscle atrophy.

The canonical signaling pathway is markedly activated in skeletal muscle during sepsis.^[^^43^^,^^70^^,^^92^^,^^96^^,^^97^^]^ Inflammatory cytokines, including IL-1β and TNF-α, upregulate SOCS box protein 1 (SPSB1) via this pathway. SPSB1 then interacts with transforming growth factor-β receptor II (TβRII) to promote its degradation, which disrupts the TβRII–Akt–Myogenin signaling axis.^[^^108^^]^ This disruption ultimately suppresses muscle protein synthesis and compromises myofiber homeostasis. Furthermore, the acute-phase protein serum amyloid A1 (SAA1) activates the TLR4–NF-κB signaling pathway.^[^^25^^,^^81^^]^ This activation induces the UPS and enhances caspase-3 expression. Together, these changes promote skeletal muscle proteolysis and accelerate the progression of SASP.

In septic skeletal muscle, the JAK-STAT signaling pathway is primarily activated by IL-6 and critically regulates inflammatory responses. IL-6 stimulation promotes activation of the transcription factor STAT3,^[^^58^^,^^93^^]^ which then induces the UPS and accelerates muscle protein degradation.^[^^104^^]^ Additionally, IL-6 may suppress protein synthesis by upregulating SPSB1, collectively exacerbating the progression of SASP.^[^^108^^]^ Animal studies indicate that STAT6 inhibition alleviates sepsis-induced skeletal muscle inflammation and improves mitochondrial function, suggesting a potential strategy for reversing SASP.^[^^45^^]^ However, current understanding faces significant limitations, as these mechanistic insights derive entirely from animal models and their relevance to human pathophysiology requires validation. Furthermore, therapeutic strategies targeting the JAK-STAT pathway remain at the preclinical stage, and their clinical translational value warrants systematic evaluation.

In septic animal models, TGF-β activates the Smad2/3 signaling pathway, which induces ROS production and activates the UPS in skeletal muscle.^[^^68^^,^^109^^]^ By inhibiting the TGF-β pathway, skeletal muscle atrophy in septic rats can be improved.^[^^76^^]^ The p38 MAPK signaling pathway exerts dual-phase effects during sepsis, initially conferring protective effects on skeletal muscle.^[^^110^^]^ Sustained TGF-β activation, however, ultimately overrides this early protection and promotes muscle atrophy via mitochondrial dysfunction.^[^[111], [112], [113]^]^

Current understanding of the inflammatory mechanisms in SASP faces significant translational challenges and fragmented evidence. Analyses of human tissue have thus far only revealed an upregulation of TLR2 and TLR4 in the vastus lateralis muscle of septic patients,^[^^25^^]^ alongside persistently elevated serum IL-6.^[^^24^^]^ In contrast, the pathological roles of other key inflammatory components, including cytokine networks such as IL-6, TNF-α, and IL-1β, and their downstream signaling pathways like NF-κB, JAK–STAT, and TGF-β, depend entirely on animal studies and lack direct in vivo validation in human skeletal muscle. Moreover, therapeutic strategies that target these pathways, including IL-6 inhibitors and JAK–STAT modulators, remain in the preclinical stage. Their safety, optimal timing of administration, and long-term efficacy in human patients have not been systematically assessed, severely hindering their clinical translation.

#### Neuromuscular junction dysfunction and excitation–contraction uncoupling

Sepsis induces significant structural and functional impairment of the neuromuscular junctions (NMJs), disrupting excitation–contraction coupling and leading to skeletal muscle dysfunction. Key pathological features include reduced acetylcholinesterase (AChE) activity, aberrant expression of postsynaptic nAChRs, dysregulated SR Ca^2+^ handling, and abnormal motor neuron discharge patterns.^[^^114^^]^ Animal studies demonstrate prolonged latency and decreased amplitude of compound muscle action potentials,^[^^55^^,^^115^^,^^116^^]^ accompanied by axonal swelling and a reduced number of NMJs.^[^^115^^]^ Furthermore, septic skeletal muscle exhibits a diminished sarcolemmal resting membrane potential,^[^^65^^]^ with some electrophysiological abnormalities persisting for up to 1 month.^[^^117^^,^^118^^]^

Under physiological conditions, nerve impulses trigger acetylcholine (ACh) release from the presynaptic membrane; ACh then binds to nAChRs on the postsynaptic membrane, inducing endplate depolarization. AChE rapidly hydrolyzes ACh to terminate signaling. Although total receptor levels may remain unchanged, aberrant expression of fetal-type nicotinic acetylcholine receptors (γ-nAChRs) and neuronal α7-type nicotinic acetylcholine receptors (α7-nAChRs) subtypes compromises signal fidelity.^[^^116^^,^[119], [120], [121]^]^ Animal studies indicate that rapamycin,^[^^119^^]^ agrin,^[^^121^^]^ and glial cell-derived neurotrophic factor^[^^116^^]^ can restore normal receptor expression and improve neuromuscular transmission. Moreover, sepsis suppresses AChE expression, and its diminished activity may be associated with protein misfolding induced by ERS.^[^^56^^]^

Even when neural transmission succeeds, sepsis severely compromises skeletal muscle contraction and relaxation. As the central organelle for calcium regulation, the SR exhibits reduced ryanodine receptors (RyRs) levels,^[^^53^^]^ which impairs calcium release and leads to weak contraction. Concurrently, decreased sarco/endoplasmic reticulum Ca^2+^ adenosine triphosphatase (SERCA) expression hinders calcium reuptake, resulting in impaired relaxation.^[^^53^^,^^122^^,^^123^^]^ Recent studies emphasize the pivotal role of ERS in sepsis-induced muscle atrophy, contributing not only to calcium dysregulation but also to the activation of apoptotic pathways that directly promote muscle fiber loss.^[^^26^^,^^58^^,^^124^^]^

Research in this field, however, faces substantial translational limitations. The structural and functional evolution of the NMJs in human sepsis, the expression profiles of key receptor subtypes, and their clinical relevance urgently require validation through well-designed clinical studies with adequate sample sizes.

### Systemic crosstalk: the vicious cycle of muscle wasting

At the organ level, multiple bidirectional vicious axes form between skeletal muscle and distant organs. Examples include suppression of hypothalamic appetite-stimulating neuropeptides, thyroid axis dysfunction, adrenal overactivation with sustained cortisol elevation, suppression of the protective ACE2–angiotensin-(1-7)-Mas receptor (ACE2-Ang-(1-7)-Mas) axis, and gut barrier disruption leading to endotoxemia. These inter-organ interactions amplify local damage and sustain a systemic state of muscle atrophy.

#### Hypothalamic suppression of appetite and energy-sensing dysregulation

The hypothalamic arcuate nucleus functions as the central hub for systemic energy metabolism. During energy deficit, the stomach releases ghrelin, a critical hunger signal, which activates the arcuate nucleus to stimulate orexigenic neuropeptides like neuropeptide Y (NPY) and agouti-related protein (AgRP) while suppressing anorexigenic neuropeptides such as pro-opiomelanocortin (POMC) and cocaine-and amphetamine-regulated transcript (CART).^[^^95^^]^ This coordinated response promotes appetite, enhances anabolism, and lowers energy expenditure. In sepsis, this energy balance network becomes profoundly disrupted.^[^^125^^]^ Diminished circulating and hypothalamic ghrelin levels reduce stimulation of the NPY/AgRP pathway, while POMC/CART expression increases.^[^^126^^,^^127^^,^^106^^]^ These changes collectively suppress appetite, accelerate protein catabolism, and impair protein synthesis, ultimately worsening skeletal muscle atrophy. Animal studies indicate that continuous light exposure aggravates this metabolic imbalance,^[^^101^^]^ whereas enteral nutrition,^[^^128^^]^ especially formulations enriched with octanoic acid,^[^^66^^]^ can elevate ghrelin, promote AgRP expression, and inhibit POMC activity, thereby partially mitigating muscle degradation. Moreover, interventions such as exogenous melatonin,^[^^129^^]^ dexmedetomidine,^[^^130^^]^ and therapeutic hypothermia ^[^^126^^]^ have been shown to reduce hypothalamic POMC levels and demonstrate potential for reversing SASP in preclinical models. However, this research area faces substantial evidence gaps and translational challenges. All current mechanistic insights into hypothalamic neuropeptides in SASP originate exclusively from animal studies. The conservation of these regulatory pathways in humans, along with the safety and efficacy of related interventions, still lacks clinical validation. Future work should prioritize confirming the pathophysiological relevance of this axis in human subjects and advancing these therapeutic strategies toward clinical application.

#### Functional thyroaxis suppression and local thyroid hormone inactivation

Septic patients frequently develop non-thyroidal illness syndrome (NTIS), a systemic condition involving dysregulated thyroid hormone metabolism. The progression of skeletal muscle atrophy, however, is primarily governed by a sharp decline in local tissue levels of active triiodothyronine (T3), which occurs earlier, is more severe, and persists longer than changes in plasma hormone levels. This local deficit largely results from the marked upregulation of type 3 deiodinase (Dio3) expression in skeletal muscle.^[^^124^^]^ During early sepsis, the pronounced induction of skeletal muscle Dio3 coincides with downregulated PGC-1α expression.^[^^39^^]^ which impairs mitochondrial biogenesis and disrupts autophagic clearance.^[^^36^^]^ These alterations collectively reduce mitochondrial content and increase the proportion of dysfunctional organelles. The expression of key antioxidant enzymes—SOD, GPX, and CAT—is also suppressed, likely due to elevated ROS levels.^[^^39^^]^ Together, these changes activate the UPS components MuRF-1 and Atrogin-1, while simultaneously inhibiting the mTOR-mediated protein synthesis pathway, thereby promoting skeletal muscle atrophy.^[^^124^^]^ Studies suggest that either targeted inhibition of Dio3^[^^124^^]^ or thyroid hormone supplementation^[^^39^^]^ can mitigate these SASP. Research in this area nevertheless remains nascent, with all current evidence derived from preclinical experiments and a complete absence of clinical validation. Because current clinical guidelines do not recommend routine thyroid hormone supplementation for septic patients, the clinical translation of this approach faces considerable obstacles.

#### Hypercortisolemia and glucocorticoid-driven muscle atrophy

Sepsis compromises the functional integrity of the hypothalamic–pituitary–adrenal (HPA) axis. Animal studies reveal that septic stress induces simultaneous hyperactivation of both the HPA axis and the sympathetic nervous system, which elevates glucocorticoid release.^[^^72^^,^^96^^,^^97^^,^^131^^]^ These hormones promote muscle weakness at the single-fiber level by upregulating the E3 ubiquitin ligases Atrogin-1 and MuRF1, thereby stimulating autophagic pathways.^[^^72^^]^ Glucocorticoids, however, exhibit dual roles, since appropriate doses of dexamethasone or hydrocortisone can suppress proteolysis and improve muscle status by modulating specific signaling pathways.^[^^113^^,^^132^^]^ Beyond classical endocrine axes, systemic inflammation and oxidative stress in sepsis dysregulate the renin-angiotensin system (RAS), leading to overactivation of the conventional angiotensin-converting enzyme–angiotensin II–angiotensin II type 1 receptor (ACE–Ang II–AT1R) axis and relative suppression of the protective ACE2–Ang-(1–7)–Mas axis. Research indicates that Ang-(1–7) supplementation mitigates SASP by inhibiting TGF-β/p38 MAPK signaling^[^^109^^,^^111^^]^ and curbing excessive autophagy.^[^^133^^]^ Insulin^[^^106^^]^ and estrogen^[^^127^^]^ have also shown protective potential in skeletal muscle. Nevertheless, this field confronts substantial limitations, as current mechanistic insights and therapeutic findings originate solely from animal studies, while human hormonal dynamics and skeletal muscle responses remain uncharacterized. Clinical data concerning dosage regimens and safety profiles for corresponding interventions are lacking, which significantly hinders translational progress.

#### Gut–muscle axis disruption: microbial dysbiosis and functional impairment in sepsis

The interplay between sepsis and the immune system involves complex mechanisms along the gut–muscle axis.^[^^134^^]^ Sepsis disrupts the intestinal mucosal barrier, induces gut microbiota dysbiosis, and subsequently impairs skeletal muscle function. Animal models of SASP demonstrate that fecal microbiota transplantation improves muscle strength and function, indicating a role for gut microbiota in SASP pathogenesis.^[^^43^^,^^132^^]^ Current hypotheses suggest that the microbiota influences muscle regeneration and homeostasis through pathways such as metabolite production, immune signaling, and altered microbial diversity.^[^^135^^,^^136^^]^ Research in this area, however, remains preliminary. The proposed mechanisms derive mainly from animal studies and correlative analyses, and their causal relevance in humans is not yet established.

## Measuring Parameters

Skeletal muscle, a key component of the musculoskeletal system, primarily generates force through contraction to drive movement. Patients with SASP frequently exhibit lower limb muscle atrophy, weakness, reduced muscle tone, diminished or absent tendon reflexes, and motor incoordination, whereas facial and ocular muscles are typically spared. These manifestations can prolong mechanical ventilation, complicate weaning, raise the risk of deep vein thrombosis, extend hospital stays, and increase mortality.^[^^137^^,^^138^^]^ Early detection and minimization of invasive diagnostics are therefore essential in sepsis management. Current clinical evaluations of skeletal muscle function encompass muscle strength testing, bedside ultrasonography (US), and computed tomography (CT). Magnetic resonance imaging (MRI) and muscle biopsy are not routinely used due to practical and clinical limitations.

### Simple assessment and biomarkers of muscle

Non-invasive approaches such as handgrip strength testing and the Medical Research Council (MRC) score are widely applied to assess peripheral muscle strength in septic patients.^[^^9^^,^^139^^,^^140^^]^ Mid-upper arm muscle circumference and triceps skinfold thickness may serve as auxiliary measures of nutritional status and muscle reserves.^[^^141^^]^ In investigating neuromuscular impairment in sepsis, skin biopsy^[^^142^^]^ and neurophysiological tests—such as sural nerve sensory nerve action potential (SNAP) and peroneal nerve testing—have been used to detect early small-fiber sensory neuropathy and associated weakness.^[^^143^^]^ More recently, easily measurable biomarkers such as plasma N-terminal pro-B-type natriuretic peptide (NT-proBNP)^[^^144^^]^ and the rate of urea creatinine ratio (UCR) change^[^^145^^]^ have emerged as potential indicators of muscle mass loss in sepsis survivors, demonstrating translational promise. These findings, however, remain preliminary and lack support from large-scale clinical studies, necessitating further validation.

### Ultrasound assessment of muscle

Bedside ultrasound, valued for its accessibility and reproducibility, has become a central tool for evaluating SASP in ICU. It enables quantitative assessment of limb muscles (such as the rectus femoris ^[^^146^^,^^147^^]^ and vastus intermedius ^[^^148^^]^) and respiratory muscles (such as the diaphragm^[^[149], [150], [151]^]^) in terms of thickness, cross-sectional area, and echogenicity. Research indicates that substantial muscle wasting occurs within the 1st week of ICU stay in septic patients,^[^^152^^]^ with rectus femoris cross-sectional area and thickness decreasing by averages of 23.2% and 17.9%,^[^^153^^]^ respectively. A 1% reduction in rectus femoris area has been linked to a 6% rise in 28-day mortality,^[^^146^^]^ underscoring its role as an independent prognostic factor. Muscle strength recovery after awakening correlates positively with muscle mass regain, and vitamin D levels may also support functional recovery.^[^^154^^]^ Combining ultrasonographic parameters with inflammatory markers holds promise for early prediction of ICU-acquired weakness.^[^^148^^]^ Regarding respiratory muscle function, diaphragmatic dysfunction is common in septic shock, and irrelevant about mechanical ventilation status.^[^^151^^]^ Recent evidence suggests that sonographic evaluation of the diaphragm and intercostal muscles—including echogenicity and thickening fraction—may help predict weaning outcomes.^[^^150^^]^ Patients who fail weaning often manifest increased respiratory muscle echogenicity, reduced intercostal thickening fraction, and more marked muscle atrophy.^[^^149^^]^ However, current evidence is constrained by small sample sizes, single-center designs, and methodological inconsistencies such as the absence of height-based normalization of muscle measurements, which limits diagnostic accuracy and generalizability. Further prospective and standardized studies are required to validate these applications.

### CT of muscle

CT remains the most extensively studied and reliable imaging modality for quantifying skeletal muscle mass in sepsis. Commonly used CT-derived metrics include psoas muscle volume at the L4 level,^[^^155^^]^ psoas linear dimensions,^[^^156^^]^ and at the L3 level: total skeletal muscle area (encompassing psoas, paraspinal, and abdominal wall muscles),^[^^157^^]^ the psoas muscle index (PMI=psoas area/height²),^[^^13^^]^ and the skeletal muscle index (SMI=total muscle area/height²).^[^^158^^]^ More recently, the pectoralis muscle index has also been introduced.^[^^159^^]^ These height-normalized indices enable more accurate assessment of muscle wasting independent of body habitus. Studies have confirmed that the PMI and SMI measured from admission CT scans effectively diagnose SASP. Reduced PMI independently correlates with 28- and 90-day mortality,^[^^160^^]^ and is significantly associated with ICU and hospital length of stay, as well as in-hospital mortality.^[^^13^^,^^14^^]^ Similarly, low SMI is linked to poorer short-term outcomes, including higher in-hospital mortality, increased incidence of multiple organ failure, greater risks of early death or chronic critical illness,^[^^160^^]^ and elevated 28-day,^[^^15^^,^^20^^]^ 90-day,^[^^158^^,^^14^^]^ and 1-year mortality.^[^^19^^]^ Although these CT-based indices are valuable, limitations remain: most supporting studies are retrospective, lack standardized diagnostic thresholds, and involve radiation exposure, which restricts repeated assessment. Prospective validation of unified protocols and cut-off values is needed before widespread clinical implementation.

Compared with CT, MRI offers superior capability in quantifying intramuscular fat, as it distinguishes between intracellular and intermuscular lipid deposition. MRI can also detect muscle edema, fibrosis, and changes in muscle contractility and elasticity, providing valuable qualitative insights into muscle health. Despite advantages such as the absence of ionizing radiation and detailed soft tissue characterization, MRI is limited in clinical practice by the lack of standardized protocols, prolonged acquisition times, complex post-processing, and undefined diagnostic thresholds. Muscle biopsy remains the most accurate method for histopathological diagnosis of myopathy.^[^^161^^]^ However, its invasive nature may impede recovery and increase the risk of secondary infection, so it is therefore not recommended for routine diagnosis of SASP.

## Therapeutic Approaches

Current management of SASP presents a significant clinical challenge, as no therapeutic strategy has been validated by large-scale randomized controlled trials. Existing clinical evidence primarily stems from small, exploratory studies that suggest potential benefits from early enteral nutrition, adequate protein-calorie support, and early mobilization (EM); however, their efficacy and feasibility require confirmation (Table 1). A pronounced translational gap characterizes this field: human studies are scarce and methodologically limited, failing to generate high-level evidence. In contrast, preclinical research has identified numerous potential therapeutic targets, including anti-inflammatory agents, antioxidants, inhibitors of protein degradation, and modulators of hypothalamic neuropeptides, yet their translation into clinical applications remains substantially hindered (Table 2). This disconnect between basic research and clinical practice severely impedes the development of effective prevention and treatment strategies.

**Table 1:** Studies of clinical treatments in patients with SASP.

Author, year	Design	Interventions groups	Sample size (control *vs.* intervention)	Interventions	Muscle sampling	Measurement	Results	Risk of bias	OCEBM Level
Wu et al., 2024^[^^181^^]^	RCT	Loading dose of 6 µg/kg for 10 min, followed by a continuous infusion at 0.2 µg/(kg · min)	22/22	Levosimendan	Diaphragm	US	Increase in diaphragm excursion and thickening fraction No effect on ventilator weaning	Moderate	1b
Abe et al., 2024^[^^165^^]^	CS	Low energy group: 810–2025 kcal Medium energy group: 2895–3873 kcal High energy group: 4334–7277 kcal	31/31/31*	7-day energy achievement rate	SMA	CT	No significant difference in 28-day mortality risk was observed among the three groups SMA decreased between CT scans in the low and medium groups but remained unchanged in the high group	Moderate	2b
Liu et al., 2022^[^^174^^]^	CS	Rehabilitation at the level of sitting on the edge of the bed or more within the first 3 days of the patients’ ICU stay	200/96	EM	-	-	Activity within the first 3 ICU days predicted better outcomes	Low	2b
Koga et al., 2018^[^^163^^]^	CS	EN was initiated within 48 h of ICU admission	56/35	EN	SMA	CT	Hospital mortality was significantly lower among patients with early EN compared to those without	Moderate	2b
Hickmann et al., 2018^[^^175^^]^	RCT	Two physiotherapy sessions per day including 30 min of continuous passive or active leg chair/bed cycling followed by 30 min manual passive or active limbs mobilization	10/8	EM	Vastus lateralis	Biopsy	Inhibition of myofibrillar protein degradation: suppressed ALP, no effect on UPS No effect on myofibrillar protein synthesis No significant alterations in inflammatory response or oxidative stress	Moderate	1b

ALP, Autophagic-lysosomal pathway; CS, Cohort study; CT, Computed tomography; EM, Early mobilization; EN, Enteral nutrition; ICU, Intensive care unit; OCEBM, Oxford Center for Evidence-Based Medicine; RCT, Randomized controlled trial; SASP, sepsis-associated sarcopenia; SMA, Skeletal muscle area at the level of the third lumbar vertebra was measured with computed tomography; US, Ultrasonography.

⁎ Sample size of low, medium and high energy groups.

**Table 2:** Preclinical studies of treatments for SASP.

Author, year	Interventions	Sample size	Timing of intervention relative to sepsis induction	Animal	Model	Treat	Muscle sampling	Period	Mechanism^⁎^	Risk of bias
Zhang et al., 2025^[^^46^^]^	Edaravone	14	After CLP	C57BL/6 mice	CLP	Single dose of 5 mg/kg, i.p.	Diaphragm	72 h	a3; c1	Unclear
Yao et al., 2025^[^^67^^]^	Melatonin	6	Before CLP	C57BL/6 mice	CLP	30 mg/(kg·d) × 7 d, i.p.	Tibialis anterior muscle	8 d	c1; e1	Unclear
Li et al., 2025^[^^64^^]^	Selenomethionine	14	After CLP	C57BL/6 mice	CLP	0.4 mg/(kg·d) × 7 d, i.p.	Gastrocnemius plantaris muscle	8 d	c1; c4; e1	Unclear
Zhang, et al., 2024^[^^66^^]^	EN + octanoic acid	10	After LPS	LPS	LPS: single dose of 5 mg/kg, i.p.	EN + 1 g/kg octanoic acid × 3 d, i.g. EN total energy intake: 100 kcal/(kg·d)	Extensor digitorum longus	3 d	c1; d2; e1; f;	Unclear
Zhang, et al., 2024^[^^102^^]^	Glutamine	6	Before LPS	Broilers	LPS: 500 µg/kg, i.p. on days 15 and 21.	Fed glutamine 21 d	Pectoralis major muscle	21 d	c1; e1	Unclear
Xiao, et al., 2024^[^^43^^]^	Vitamin K1	10	After LPS	C57BL/6 mice	LPS: single dose of 15 mg/kg, i.p.	Single dose of 1 mg/kg, i.g.	Gastrocnemius	24 h	a3; e2	Unclear
Wang, et al., 2024^[^^47^^]^	Anisodamine	7–8	Before CLP	C57BL/6 mice	CLP	Single dose of 0.02 mg/kg, i.p.	Diaphragm	72 h	a3; c1	Unclear
Li,et al., 2024^[^^42^^]^	Whole milk protein powder	12	Before LPS	C57BL/6 mice	LPS: single dose of 15 mg/kg, s.c.	3 g/(kg·d) × 20 d, i.g.	Quadriceps Gastrocnemius	21 d	a3; a4; c1; d2; e1	Unclear
Chen,et al., 2024^[^^57^^]^	N-Acetylcysteine	8	Before and after CLP	C57BL/6 mice	CLP	1 g/kg at 1 h pre-operation and at 24 h and 48 h post-operation, i.g.	Tibialis anterior	72 h	b;c1	Unclear
Hou et al., 2023^[^^85^^]^	Leucine	20	After CLP	C57BL/6 mice	CLP	0.1 g/(kg·d) × 4 d, i.p.	Gastrocnemius	4 d	c3; d1; d2; e1	High^†^
Dai et al., 2023^[^^68^^]^	Astragaloside IV	12	Before and after CLP	C57BL/6 mice	CLP	20 mg/kg at 1 h, 24 h, and 48 h post-operation, i.g.	Tibialis anterior muscle	72 h	c1; e1; e3; e4	High^†^
Yin et al., 2022^[^^75^^]^	Neuregulin-1β	7	After CLP	SD rats	CLP	10 µg/(kg 12 h) × 6 doses, i.v.	Tibialis anterior	72 h	c2; d2	Unclear
Duan et al., 2022^[^^129^^]^	Melatonin	6	After LPS	SD rats	LPS: single dose of 10 mg/kg, i.p.	30 mg/(kg·d) × 3 d, i.p.	Gastrocnemius	3 d	c1; f	Unclear
Cao et al., 2022^[^^95^^]^	EN	6	After LPS	SD rats	LPS: single dose of 5 mg/kg, i.p.	EN 100 kcal/(kg·d) × 3 d, i.g.	Extensor digitorum longus	3 d	c1; d2; f	High^‡^
Tsuji et al., 2021^[^^34^^]^	Farnesyltransferase inhibitor	5	After CLP	CD1 mice	CLP	5 mg/(kg·d) × 3 d,i.g.	Soleus Gastrocnemius	3 d	a1; a2	Unclear
Supinski et al., 2021^[^^49^^]^	Mitoquinone mesylate	5–6	After CLP	CD1 mice	CLP	3.5 mg/(kg·d) × 2 d,i.p.	Diaphragm	48 h	a3; a4	Unclear
Li et al., 2021^[^^73^^]^	Enoxaparin	5	After LPS and before mechanical ventilation	C57BL/6 mice	LPS: single dose of 1 mg/kg, i.v.+ mechanical ventilation	Single dose of 4 mg/kg, i.v.	Diaphragm	8 h	a1; a3; c1-c3; e1	Unclear
Lee, et al., 2021^[^^172^^]^	Lithium chloride	3	Before LPS	C57BL/6 mice	LPS: single dose of 1 mg/kg, i.p.	40 mgc/(kg·d) × 4 d	Quadriceps	4 d	c1; e1	Unclear
Wang et al., 2020^[^^182^^]^	Testosterone propionate	8	After CLP	SD rats	CLP	10 mg (kg· 3.5 d) × 6 times, s.c.	Extensor digitorum longus	3 weeks	d2	Unclear
Supinski et al., 2020^[^^77^^]^	MitoTEMPOL	5–6	After CLP	CD1 mice	CLP	Single dose of 10 mg/kg, i.p.	Diaphragm	48 h	a4; c3; c4	Unclear
Jiang et al., 2020^[^^183^^]^	Magnesium-sulfate	10	After CLP and mechanical ventilation	SD rats	CLP+ mechanical ventilation	27 mg/(kg·h) × 10 h, i.v.	Diaphragm	18 h	e1; e2	Unclear
Irahara et al., 2020^[^^180^^]^	NMES	10–12	After LPS	C57BL/6 mice	LPS: single dose of 20 mg/kg, i.p.	Bilateral gastrocnemius muscle for 1 h at frequency (LF=2 Hz) and voltage (LV=10 V) × 2 times	Gastrocnemius	72 h	a2; e1	High^†^
Habr et al., 2020^[^^113^^]^	Hydrocortisone	10	After CLP	Wistar rats	CLP	3 mg/(kg·d) × 5 d, i.p.	Triceps brachii	5 d	e5	Unclear
Wu et al., 2019^[^^184^^]^	Neuregulin-1β	10	After CLP	SD rats	CLP	10 µg/(kg· 12 h) × 6 times, i.v.	Diaphragm	72 h	d2	Unclear
Yu et al., 2018^[^^173^^]^	Heme oxygenase-1	5	Before CLP	C57BL/6 mice	CLP	Single dose of 50 mg/kg, i.p.	Solei	24 h	a3; c1; e1	Unclear
Martín et al., 2018^[^^96^^]^	Formoterol	10	After LPS	Wistar rats	LPS:25 µg/kg × 2 times, i.p.	50 µg/kg × 2 times, i.p.	Gastrocnemius	19 h	c1; c2; d2; e1; e2	Unclear
Zhang, et al., 2017^[^^37^^]^	Hydrogen sulfide	6	After CLP	SD rats	CLP	Single dose of 50 µmol/kg, i.p.	Diaphragm	24 h	a1; a3 a4;	Unclear
Cheng et al., 2017^[^^130^^]^	Dexmedetomidine	12	After LPS	SD rats	LPS: single dose of 5 mg/kg, i.p.	50 µg/kg, i.v.	Extensor digitorum longus	24 h	c1; e1; f	Unclear
Ozkok et al., 2016^[^^54^^]^	Melatonin	8	Before and after LPS	Wistar Albino rats	LPS: single dose of 20 mg/kg, i.p.	10 mg/kg × 3 times, i.p.	Gastrocnemius	8 h	a3; a4	Unclear
Ham et al., 2016^[^^105^^]^	Glycine	15	Before LPS	C57BL/6 mice	LPS: single dose of 1 mg/kg, i.p.	Single dose of 1 mg/kg, i.p.	Quadriceps	5 h	a3; e1	Unclear
Wang et al., 2016^[^^169^^]^	Asparagine	6	Before LPS	Barrows	LPS: single dose of 100 µg/kg, i.p.	Fed 1.0% asparagine × 17 d	Gastrocnemius	17 d	c1; e1; g	Unclear
Zhang et al., 2015^[^^131^^]^	Alanyl-glutamine	6	Before and after LPS	Barrows	LPS: 100 mg LPS/kg × 2 times	Fed alanyl-glutamine × 10 d	Skeletal muscle	15 d	c1; d2	Unclear

ALP, Autophagic-lysosomal pathway; ATP, Adenosine triphosphate; CLP, Cecal ligation and puncture; EN, Enteral nutrition; ERS, Endoplasmic reticulum stress; i.g., Intragastric administration; i.p., Intraperitoneal injection; i.v., Intravenous injection; JAK-STAT, Janus kinase-signal transducer and activator of transcription; LPS, Lipopolysaccharide; MuSCs, Muscle satellite cells; mTOR; Mammalian target of rapamycin; MQC, mitochondrial quality control; NF-κB, Nuclear factor kappa-B; NMES, Neuromuscular electrical stimulation; POMC, Pro-opiomelanocortin; P38 MAPK, p38 mitogen-activated protein kinase; s.c., Subcutaneous injection; SD, Sprague–Dawley rats; TGF-β, Transforming growth factor-β; TLR, Toll-like receptor; UPS, Ubiquitin–proteasome system.

^⁎^a1: Improved mitochondrial morphology; a2: Enhanced mitochondrial MQC; a3: Suppressed mitochondrial oxidative stress; a4: Increased mitochondrial ATP synthesis; b: Inhibited ERS; c1–c3: Suppressed myofibrillar degradation via UPS, ALP, and calpain, respectively; c4: Inhibited programmed cell death (pyroptosis/apoptosis); d1: Activation and differentiation of MuSCs; d2: Stimulated protein synthesis (mTOR pathway); e1: Inhibited excessive TLR and cytokine production; e2–e5: Suppressed myofibrillar degradation (NF-κB, JAK-STAT, TGF-β, P38 MAPK pathways); f: Suppressed hypothalamic POMC via ghrelin; g: Attenuated excessive serum cortisol.

High^†^: ≥5 unclear risk biases; High^‡^: ≥1 high-risk bias.

### Nutritional support

Skeletal muscle mass homeostasis depends on a dynamic balance between protein synthesis and degradation. SASP is pathologically characterized by net myofiber degradation. Reversing this process requires a dual approach that promotes protein synthesis while inhibiting excessive catabolism, nutritional interventions show significant potential through their timing and composition.

Current clinical guidelines recommend initiating enteral nutrition (EN) as early as possible after hemodynamic stabilization in septic patients.^[^^162^^]^ Among patients with pre-existing sarcopenia, EN initiated within 48 h of admission correlates with lower in-hospital mortality (OCEBM Level 2b).^[^^163^^]^ This strategy aligns with recommendations from the French Intensive Care Society (SRLF) and the European Society for Clinical Nutrition and Metabolism (ESPEN), which advocate for early nutritional support within 48 h of ICU admission, with EN being the preferred route in the absence of contraindications.^[^^162^^,^^164^^]^ Furthermore, retrospective studies suggest that adequate energy supply may reduce the risk of sarcopenia in this population (OCEBM Level 2b).^[^^165^^]^ However, there are currently no specific guidelines recommending the use of particular tools for calculating energy requirements in critically ill patients.^[^^164^^,^^166^^]^ Existing consensus tends to favor early low-calorie feeding strategies to avoid potential overfeeding associated with high-calorie intake.^[^^162^^]^ Therefore, whether a high-calorie nutritional regimen should be adopted early in sepsis patients remains controversial and requires further clinical evidence. The above conclusions are primarily derived from small-sample, retrospective studies with potential residual confounding biases, highlighting the need for prospective studies for validation.

Mechanistic studies indicate that EN,^[^^95^^]^ particularly when combined with formulations such as octanoic acid,^[^^66^^]^ can elevate circulating ghrelin levels, thereby suppressing hypothalamic POMC neuron overactivation and ameliorating muscle atrophy in animal models. Amino acid metabolism is critically involved in SASP.

Clinical observations reveal that higher levels of branched-chain amino acids (BCAAs) are independently associated with reduced ICU and 28-day mortality, although the mechanisms remain unclear.^[^^167^^]^ Small interventional studies propose that amino acid supplementation during the acute inflammatory phase may activate the mTORC signaling pathway, promoting protein synthesis and reducing systemic and muscular protein loss in sepsis.^[^^168^^]^ Still, these clinical insights remain preliminary and await confirmation through large-scale randomized trials. Regarding nutritional composition, preclinical studies indicate that supplements including selenomethionine,^[^^64^^]^ leucine,^[^^85^^]^ asparagine,^[^^169^^]^ and alanyl-glutamine^[^^169^^]^ can alleviate SASP through multiple pathways—such as anti-inflammatory effects, proteolysis suppression, and oxidative stress attenuation. Currently, clinical guidelines do not recommend routine supplementation of specific amino acids for patients with sepsis. The consideration of glutamine supplementation is only mentioned for patients with trauma or burns.^[^^162^^]^ Therefore, there is insufficient evidence to support the need for additional amino acid supplementation in sepsis patients complicated with sarcopenia, and further research is required to clarify this issue.

Increasing dietary fat has been proposed to reduce protein breakdown by favoring lipid oxidation for energy during septic stress, though its clinical safety and efficacy require systematic evaluation. Whole milk protein powder has also demonstrated potential in ameliorating muscle atrophy in animal experiments.^[^^42^^]^ In recent years, novel strategies such as ketogenic diets^[^^170^^]^ and GLP-1 receptor agonists^[^^171^^]^ have been proposed as potential interventions. However, these hypotheses currently lack mechanistic or clinical substantiation. In summary, while nutritional interventions targeting timing and composition hold theoretical promise, the current evidence remains limited by small-scale clinical studies, insufficient mechanistic insight, and a lack of validation for innovative approaches. Future research should prioritize well-designed, large-scale clinical trials with clearly defined mechanisms to advance personalized nutritional therapy for SASP.

### Drugs and targeted therapy

Based on current insights into the pathogenesis of SASP, a multi-level framework has been established to explore potential drugs and targets directed at key pathological processes. Organelle-level investigations primarily address the clearance of dysfunctional mitochondria and the suppression of excessive oxidative stress. At the cellular level, the central strategy involves inhibiting hypercatabolism of protein mediated by the UPS and ALP. Research on the tissue level aims to reduce the expression of inflammatory cytokines such as IL-6, TNF-α, and IL-1β, thereby attenuating their contribution to muscle protein degradation. On the organ-interaction level, many interventions seek to ameliorate skeletal muscle atrophy by modulating hypothalamic appetite-related neuropeptides. However, fundamental studies in this area are generally constrained by small sample sizes, variability in experimental subjects, and the use of different modeling methods—mainly cecal ligation and puncture (CLP) and intraperitoneal lipopolysaccharide (LPS) injection. These methodological differences hinder cross-study comparisons and the integration of experimental results. Furthermore, the translational potential of related targeted drugs, including their feasibility, safety, and effective dosage ranges, requires systematic evaluation. A more significant limitation concerns the timing of drug administration in current intervention studies. Agents such as melatonin,^[^^67^^]^ anisodamine,^[^^47^^]^ enoxaparin,^[^^73^^]^ lithium chloride,^[^^172^^]^ and heme oxygenase-1^[^^173^^]^ are frequently administered prior to sepsis induction. Such pretreatment strategies have limited clinical applicability, as the risk–benefit ratio of pharmacologically preventing sarcopenia in patients without a sepsis diagnosis remains unclear. Additionally, the technical challenges associated with muscle biopsy in clinical settings restrict mechanistic analysis using human samples and impede the precise development of targeted therapies. Future efforts should prioritize the establishment of non-invasive biomarker systems and the implementation of appropriately timed interventional trials to effectively translate basic mechanistic discoveries into clinical practice.

### Early rehabilitation treatment

EM and neuromuscular electrical stimulation (NMES) are central to the rehabilitation of patients with sepsis and septic shock. Current evidence suggests that EM helps alleviate skeletal muscle atrophy and reduces long-term muscle wasting caused by disuse in these patients. A retrospective study found that achieving EM within the first 3 days of ICU admission significantly improved patient outcomes (OCEBM Level 2b).^[^^174^^]^ Another small clinical study proposed that this protective effect might stem from suppressed myofiber autophagy, although it observed no significant inhibition of the UPS or increase in myofiber protein synthesis (OCEBM Level 1b).^[^^175^^]^ Animal studies further corroborate the benefits of early exercise in sepsis models.^[^^176^^]^ However, existing research is still limited, and both the underlying mechanisms and clinical applicability warrant systematic investigation. Evidence for NMES currently comes mainly from subgroup analyses of clinical trials and small-sample case reports. Preliminary findings indicate that the technique is safe for septic patients,^[^^177^^]^ enhances compound muscle action potentials^[^^152^^]^ and muscle strength,^[^^178^^]^ and may produce better outcomes in individuals with higher baseline functional status.^[^^179^^]^ Still, no large-scale clinical trials have confirmed its efficacy, and research in this area remains preliminary. Basic studies indicate that its protective mechanisms may involve attenuated inflammatory responses and improved mitochondrial function.^[^^180^^]^ In summary, while both EM and NMES hold therapeutic promise, the current evidence is limited by small study sizes, incomplete mechanistic insight, and insufficient clinical validation. Future research should prioritize well-designed, large-scale clinical studies to clarify their mechanisms, identify suitable patient groups, and define optimal treatment protocols.

## Conclusions

SASP is a severe complication that significantly impacts the long-term prognosis of patients. This condition often progresses in parallel with multiple organ dysfunction, clinically manifesting as muscle failure characterized by progressive muscle atrophy, decreased muscle strength, and functional loss. Currently, there is no standardized clinical assessment system for SASP. Although CT is the most commonly used and reliable method for assessing muscle mass, its operational complexity and cost limit its routine clinical application. Bedside muscle strength testing is simple and feasible but lacks sufficient sensitivity. Therefore, developing molecular biomarkers and early diagnostic methods with high sensitivity, high specificity, and clinical feasibility is an important direction for future research. Bioelectrical impedance analysis (BIA) is currently used in clinical practice to assess skeletal muscle mass in patients, and its potential application in SASP assessment also warrants further exploration. From a pathophysiology perspective, SASP is a cumulative process: it begins with organelle dysfunction, which is amplified through multi-level signaling cascades, ultimately leading to multi-organ damage. Most current mechanistic studies remain at the preclinical stage, and the clinical translation of antioxidant or anti-inflammatory drugs targeting key signaling pathways has shown limited effectiveness. Clinical interventions also face limitations: methods such as early EN, bedside rehabilitation, and NMES have been preliminarily confirmed as safe and somewhat effective, but related studies are generally small in scale, lack statistical power, and lack strong evidence-based recommendations. Future research should focus on the following two directions: At the basic research level, further elucidation of the molecular networks and inter-organ interaction mechanisms underlying the onset and progression of sarcopenia is needed to accelerate the translation of promising targeted therapeutic strategies into clinical trials. At the clinical practice level, greater emphasis should be placed on sarcopenia screening and skeletal muscle function protection during the early stages of sepsis. There is an urgent need to conduct well-designed, large-scale interventional clinical studies to establish prevention and treatment strategies based on high-quality evidence. Ultimately, the core goals of comprehensive sepsis management should include improving patients’ long-term quality of life and promoting functional recovery (Figure 6).

**Figure 6:**
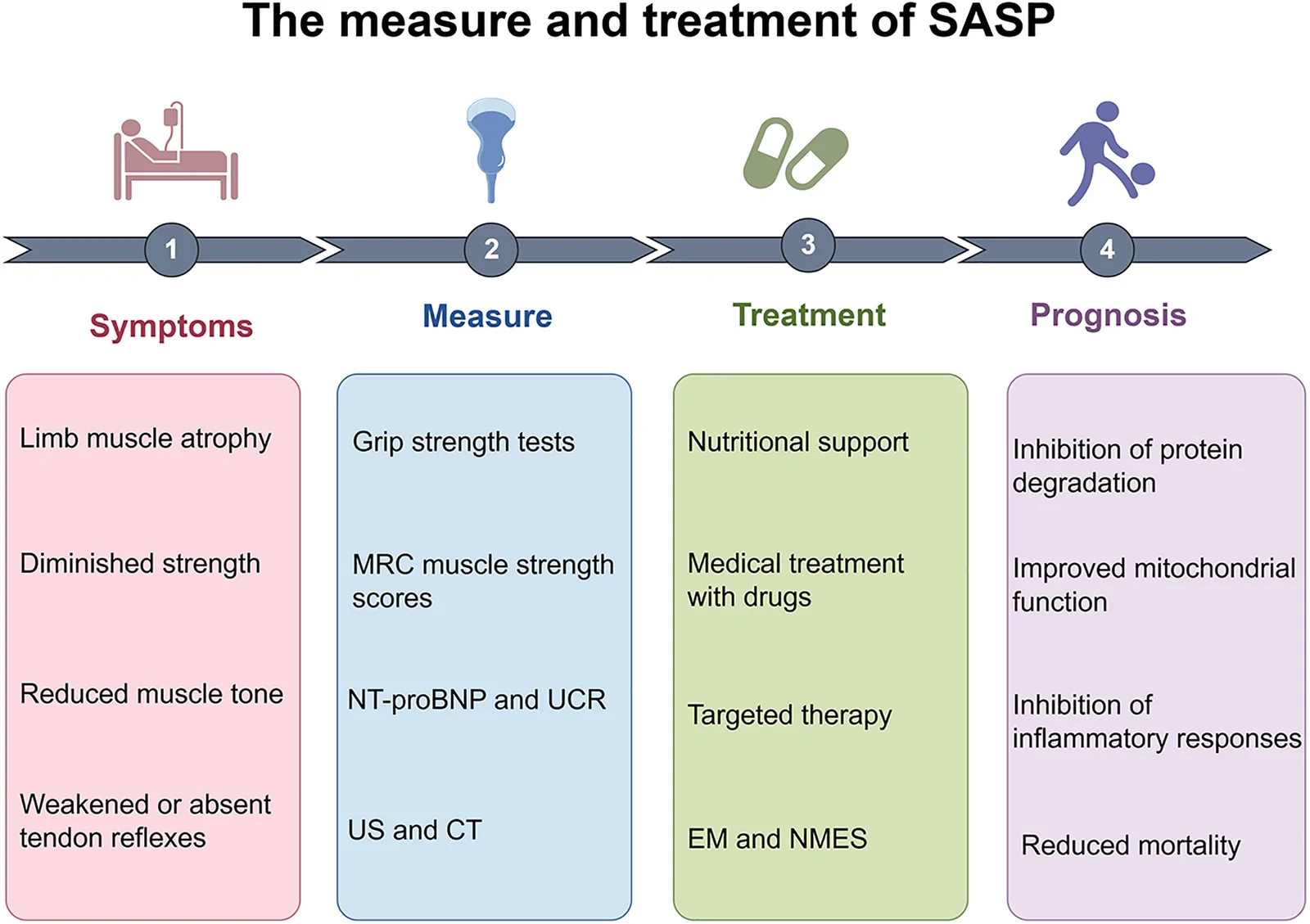
The measure and treatment of SASP. Research on SASP focuses on its symptoms, measure, and treatment to ultimately improve patient prognosis. The primary clinical manifestations are limb muscle atrophy, diminished muscle strength, reduced muscle tone, and weakened or absent tendon reflexes. Current non-invasive assessments include grip strength tests and the MRC muscle strength scores. Emerging biomarkers such as NT-proBNP and UCR show promise, while imaging relies primarily on US and CT. Treatment strategies encompass nutritional support, mechanism-based drugs and targeted therapy, EM, and NMES. These approaches collectively aim to reduce mortality by inhibiting myofibrillar protein degradation, improving mitochondrial function, and inhibiting inflammatory responses. This integrated framework guides the contemporary assessment and management of SASP. CT, Computed tomography; EM, Early mobilization; MRC, Medical Research Council; NMES, Neuromuscular electrical stimulation; NT-proBNP, N-terminal pro-B-type natriuretic peptide; SASP, Sepsis-associated sarcopenia; UCR, Urea creatinine ratio; US, Ultrasonography.

## Acknowledgments

Figure 1, Figure 2, Figure 3, Figure 4, Figure 5, Figure 6 were created with figdraw.com.

## Funding

This research was funded by the Sichuan Provincial Administration of Traditional Chinese Medicine Scientific Research Project of Traditional Chinese Medicine (2024MS190); Sichuan Provincial Healthcare Research Project for Officials (Chuanganyan 2023-208); and Sichuan Provincial Science and Technology Program (2023YFS0134).

## Ethics Statement

Not applicable.

## Conflict of Interest

The authors declare that they have no known competing financial interests or personal relationships that could have appeared to influence the work reported in this paper.

## Data Availability

Not applicable.

## CRediT authorship contribution statement

**Xiujun Chang:** Writing – review & editing, Writing – original draft. **Fan Zeng:** Writing – review & editing, Writing – original draft. **Jun Wang:** Writing – review & editing. **Zhaoxuan Guo:** Writing – review & editing. **Rongan Liu:** Writing – review & editing. **Yu Lei:** Writing – review & editing. **Jiajia Li:** Writing – review & editing. **Fuxun Yang:** Writing – review & editing. **Xiaoxiu Luo:** Writing – review & editing. **Chun Pan:** Writing – review & editing. **Xiaobo Huang:** Writing – review & editing. **Yunping Lan:** Writing – review & editing, Writing – original draft, Funding acquisition.
